# Design and Static Analysis of MEMS-Actuated Silicon Nitride Waveguide Optical Switch

**DOI:** 10.3390/mi16080854

**Published:** 2025-07-25

**Authors:** Yan Xu, Tsen-Hwang Andrew Lin, Peiguang Yan

**Affiliations:** 1 College of Physics and Optoelectronic Engineering, Shenzhen University, Shenzhen 518060, China; yanpg@szu.edu.cn; 2 Shenzhen Lighting Institute, Shenzhen 518055, China; andrew.lin@szli.org

**Keywords:** photonics integrated circuit, silicon photonics, microelectromechanical system, optical switch

## Abstract

This article aims to utilize a microelectromechanical system (MEMS) to modulate coupling behavior of silicon nitride (Si_3_N_4_) waveguides to perform an optical switch based on a directional coupling (DC) mechanism. There are two states of the switch. First state, a Si_3_N_4_ wire is initially positioned up suspended in the air. In the second state, this wire will be moved down to be placed between two arms of the DC waveguides, changing the coupling behavior to achieve bar and cross states of the optical switch function. In the future, the MEMS will be used to move this wire down. In this work, we present simulations of the two static states to optimize the DC structure parameters. Based on the simulated results, the device size is 8.8 μm × 55 μm. The insertion loss is calculated to be approximately 0.24 dB and 0.33 dB, the extinction ratio is approximately 24.70 dB and 25.46 dB, and the crosstalk is approximately −24.60 dB and −25.56 dB, respectively. In the C band of optical communication, the insertion loss ranges from 0.18 dB to 0.47 dB. As such, this device will exhibit excellent optical switch performance and provide advantages in many integrated optics-related optical systems applications. Furthermore, it can be used in optical communications, data centers, LiDAR, and so on, enhancing important reference value for such applications.

## 1. Introduction

As one of the components in photonic integrated circuits (PICs), the optical switch can be compared to transistor in ICs and plays a vital role in optical communications [1,2], data centers [3,4,5,6,7], LiDAR [8,9,10], and other fields. So far, on-chip optical switches have been researched for decades. In terms of mechanisms, the main ones include thermo-optic and electro-optic effects. In terms of structures, the main ones include the Mach–Zehnder interferometer (MZI), microring resonator (MRR), etc. Thermo-optic switches based on the MZI, which combine subwavelength gratings to enhance bandwidth, have their designed switching cell with an extinction ratio of about 13 dB, an insertion loss of less than 2 dB, and crosstalk of 12 dB, over a bandwidth of 150 nm, as well as a footprint of 240 μm × 9 μm [11]. However, heating and heat dissipation easily result in low switching speeds, such as in [12], where, for a 1 × 8 thermo-optic switch based on a silica waveguide with a footprint of 18,669 × 1754 μm^2^, the measured insertion loss is less than 3.69 dB, the rise time and fall time are 1.0 ms and 1.24 ms, respectively, and the average power is 1830 mW. In [13], a polymer thermo-optic switch based on MZI achieved an insertion loss of 2.6 dB with a driving power of 4.5 mW; the rise and fall times of the device are 400 μs and 600 μs, respectively. Electro-optic switches can achieve switching times on a nanosecond scale due to carrier injection. However, the presence of carriers leads to the absorption of photon energy, resulting in larger insertion losses compared to thermo-optic switches. In [14], a 2 × 2 silicon electro-optic switch unit based on a double ring-assisted MZI has experimentally demonstrated insertion losses of 1.8 dB to 3.4 dB, and the rise and fall times of the switch are 405 ps and 414 ps, respectively. In particular, the optical bandwidth of electro-optic switches based on MRRs is very narrow, and it is usually intended for certain narrow-bandwidth requirements. For example, in [15], the optical bandwidth is 0.09 nm and fast switching speeding is 10 ns. Therefore, both types of optical switches have various limitations when integrated on a large scale on a chip. The modulating mechanism of a MEMS-actuated waveguide optical switch utilizes mechanical movement of the microstructure. Its advantages are that it does not rely on the thermo-optic effect or electro-optic effect, that is, there is no heating and heat dissipation, nor it is affected by carriers, and the switching time of electrostatic actuation can be at a microsecond scale [16,17,18,19,20]. Through low-loss material, high switching speed and low insertion loss can be simultaneously achieved; actuated component can be integrated in a waveguide layer and compatible with PIC technology [21]; there is no power consumption in the static state [22,23]; etc. Therefore, research on MEMS-actuated waveguide optical switches has substantial engineering application value.

The required performance for optical switches mainly includes a compact footprint, certain range of bandwidth, large extinction ratio, low crosstalk, low insertion loss, high switching speed, low power consumption, etc., when they are applied in an N × N switch array. For optical communications or LiDAR, insertion loss, extinction ratio, crosstalk, broad bandwidth, power consumption, and switching speed are critical. Especially for LiDAR, the compact footprint of the switch unit enables the construction of finer scanning for objects. In 2002, Sandia National Laboratories in the United States built a polymer waveguide based on MEMS, with comb-shaped MEMS actuators on both sides of the waveguide. The input waveguide was moved laterally by electrostatic force to align with any of two outputs, thereby switching the optical path. However, perfect alignment was difficult, easily resulting in large coupling losses [24]. Subsequently, the United States and Japan have successively carried out related research, using comb-shaped MEMS actuator structure and the same mechanism, but none of them were satisfactory [25,26,27]. In 2018, Tohoku University in Japan designed a lateral comb-actuated adiabatic coupling optical switch with a port isolation of 16.7 dB, insertion loss less than 1 dB, and switching time of 36.7 μs. But three actuators were required to control the coupling gap of a pair of silicon waveguides to achieve optical switching. The device structure was complex and large in size [20]. The Korea Advanced Institute of Science and Technology improved to the point where a MEMS actuator could be used to control change in the waveguide coupling gap. The switch has a 59 dB extinction ratio, insertion loss less than 4.0 dB, a footprint of 1.2 × 4.5 mm^2^, switching time of 9.8 μs, switching voltage of 9.6 V, and 20 dB bandwidth of 31.5 nm. But it also uses lateral control and still occupies a large space [19]. Currently, the most advanced MEMS-actuated waveguide optical switch comes from the University of California, Berkeley. In 2016, a vertical adiabatic coupler silicon optical switch with 42 V pull-in voltage was designed, which changed the optical path by adjusting the vertical coupling distance of the upper and lower straight waveguides, and then a 64 × 64 optical switch array was constructed. The 64 × 64 digital silicon photonic switch has a 3.7 dB on-chip insertion loss and broadband operation of 300 nm. The measured switching time is 0.91 μs, and the extinction ratio is larger than 60 dB [17]. In 2019, an on-chip 240 × 240 switch matrix was prepared, which is currently the world’s largest silicon-based MEMS technology optical switch array as far as we know [28]. In 2022, the above optical switch was used as routing switches for “row” and “column” path selection to control a 2D beam-scanning system, to demonstrate a LiDAR system with focal plane optics together with a 128 × 128 solid-state frequency modulated continuous wave (FMCW) and to reconstruct an image combining the light source and the returned beam [10]. In particular, the University of California, Berkeley achieved “CMOS + PIC + MEMS” chip-level integration and preliminarily completed verification of solid-state LiDAR with a closed-loop “control + logic + actuation” function.

In this paper, we modulate the coupling between silicon nitride (Si_3_N_4_) waveguides to perform an optical switch based on a directional coupling (DC) structure, changing the position of the microstructure, switching optical paths, and realizing the switching function. The switch is characterized by the following: its waveguide core layer uses Si_3_N_4_ material, which is compatible with PIC technology and enables a compact footprint; the Si_3_N_4_ material has no nonlinear absorption effect, resulting in low insertion loss of the device; there is a small insertion loss variation in the communication C band; it adopts an electrostatic force-actuated microstructure mechanism, which requires power only during actuation, thus easily achieving low power consumption. Here, a new design of an optical switch and the analysis of its static optical properties are the key focuses in this paper.

## 2. Mechanism of the Optical Switch

**Figure 1:**
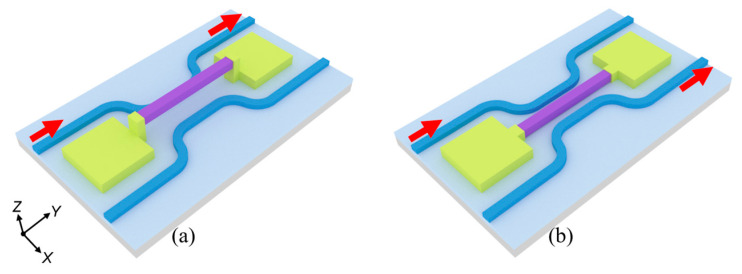
Schematic diagram of the proposed optical switch: (**a**) bar state; (**b**) cross state.

**Figure 2:**
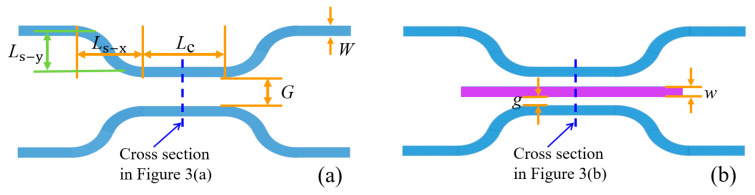
Top view of the optical switch: (**a**) Bar state, (**b**) Cross state.

**Figure 3:**
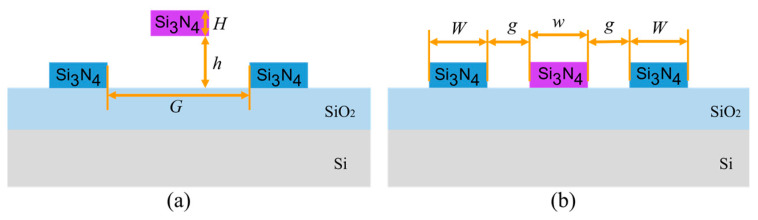
Cross-section coupling area of the optical switch: (**a**) bar state; (**b**) cross state.

The structure of the optical switch is shown in Figure 1. The optical switch is based on a DC structure, which consists of DC waveguides and a straight waveguide. The waveguide core layer is Si_3_N_4_, with a refractive index of 2 at wavelength of 1550 nm. The substrate is silicon (Si), and the lower and upper cladding layers are silicon dioxide (SiO_2_) and air, respectively, with refractive indices of 1.44 and 1, respectively. The purple waveguide in the middle of the DC is a straight waveguide that affects optical coupling. The yellow squares are simplified MEMS actuators. The basic principle of the optical switch is that when the straight waveguide is suspended, the distance between two DC waveguides is large, resulting in the inability of light to couple to the other waveguide, presenting the bar state; when the straight waveguide is pulled down by the MEMS actuators, optical coupling will occur in the DC straight waveguide coupling area, realizing optical path changing, presenting the cross state. A schematic diagram of the corresponding top-view structure is shown in Figure 2, and a schematic diagram of a cross-section of the coupling area is shown in Figure 3.

## 3. Parameter Optimization and Optical Performance Analysis

**Figure 4:**
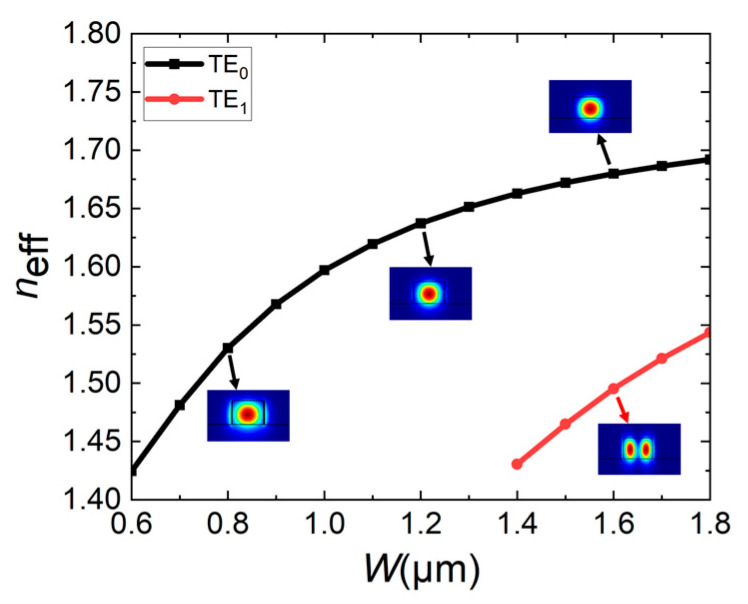
When waveguide height *H* = 0.5 μm, the effective refractive indices *n*_eff_ of waveguide varies with waveguide width *W*, and the insets show the mode fields at corresponding sizes.

Here, the optical performance of the proposed switch is analyzed. To more accurately analyze the device performance, the three-dimensional finite-difference time-domain (3D FDTD) method is utilized for optimization, with a mesh of 0.02 µm in the *X* direction, 0.05 µm in the *Y* direction, and 0.01 µm in the *Z* direction. Considering the calculated capacity of the computer, the waveguide roughness is set to zero. In order to achieve better performance with size reduction so as to only work under the fundamental mode, the single-mode waveguide is calculated. With an incident wavelength of 1.550 μm and waveguide height *H* of 0.5 μm as an example, the effective refractive indices *n*_eff_ under different waveguide widths *W* are calculated as shown in Figure 4, and the insets are the mode fields under the corresponding size.

It is found that when *W* < 1.4 μm, the mode supported by the waveguide is still only the fundamental mode TE_0_; when *W* ≥ 1.4 μm, the high-order mode TE_1_ appears. In order to reduce device size, the waveguide width *W* is selected to be 0.8 μm. Considering factors such as ideal coupling, simplified structure, and reduced fabrication difficulty, the size of the middle straight waveguide, 0.8 μm (*w*) × 0.5 μm (*H*), is selected.

**Figure 5:**
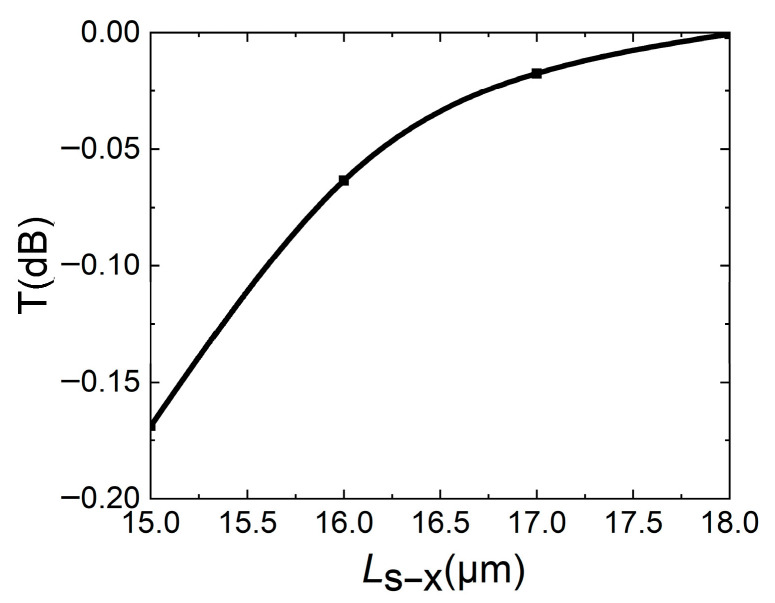
When *L*_s-y_ = 2.9 μm, the transmission T of bending waveguide is related to *L*_s-x_.

After the basic size of the waveguide is determined, in order to reduce the insertion loss of the device, the bend length of the DC curved waveguide needs to be determined. For the top view of the DC structure in Figure 2a, it can be seen that *L*_s-y_ and *L*_s-x_ jointly affect the bending degree of the waveguide. In order to simplify calculation and reduce device size, when *L*_s-y_ is selected as 2.9 μm, the relationship between transmission T of waveguide and *L*_s-x_ is calculated, and the result is shown in Figure 5. As shown in Figure 5, as *L*_s-x_ increases, the transmission of the waveguide increases. That is, as *L*_s-x_ increases, the curvature of the waveguide becomes gentler, and the mode in the waveguide changes less. Considering the device size, when *L*_s-y_ = 2.9 μm, *L*_s-x_ is selected as 17 μm.

**Figure 6:**
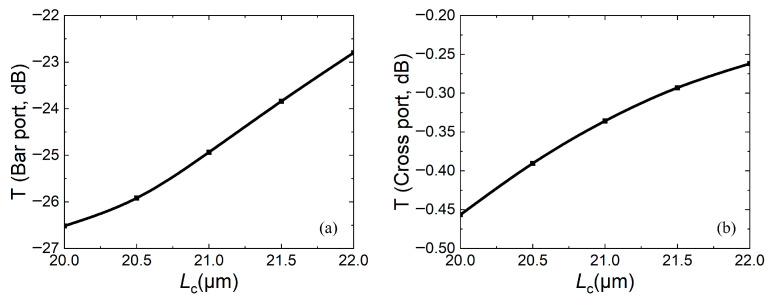
When *g* = 0.25 μm and moving down straight waveguide, relationship between transmission and coupling length *L*_c_: (**a**) bar port; (**b**) cross port.

For DC structures, the key structural parameters that affect DC coupling are coupling length and coupling gap. The coupling gap should not be too small and should be selected based on the characteristic size of lithography. Otherwise, the difficulty of fabrication will increase and the fabrication parameters will not easily meet the theoretical design, thereby affecting performance. For the structural parameters in Figure 2, since the waveguide width *W* has been determined to be 0.8 μm, considering reducing the subsequent difficulty of static fabrication and device size, *g* is selected as 0.25 μm, that is, *G* = 2*g* + *w* = 1.3 μm. For a coupling gap of 1.3 μm in a DC structure, it is difficult to achieve optical coupling, so the coupling length needs to be determined under the condition that the suspended straight waveguide is pulled down. In Figure 6, the relationship between transmissions of the bar port and cross port and the coupling length *L*_c_ when *g* = 0.25 μm is calulated. Since the straight waveguide is moved down, the larger the transmission of the cross port, the better the performance. It can be seen that the transmissions of both ports increase with increasing coupling length. This is because different coupling lengths lead to different light energies in the straight waveguide, which in turn affects the energy of two output ports.

**Figure 7:**
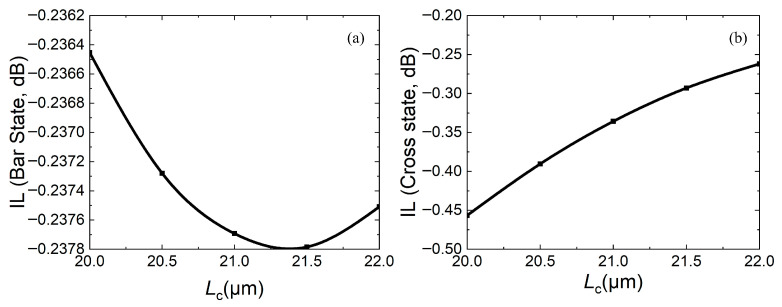
Relationship between insertion loss IL and coupling length *L*_c_: (**a**) bar state; (**b**) cross state.

**Figure 8:**
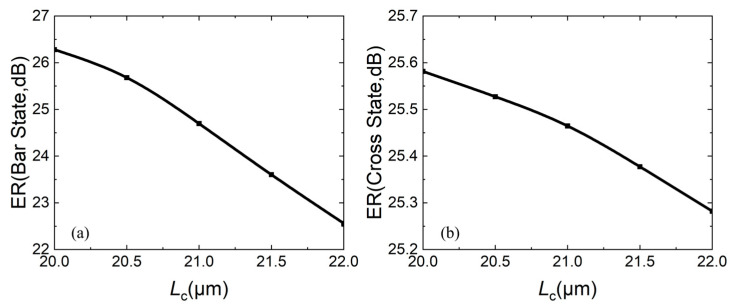
Relationship between extinction ratio ER and coupling length *L*_c_: (**a**) bar state; (**b**) cross state.

**Figure 9:**
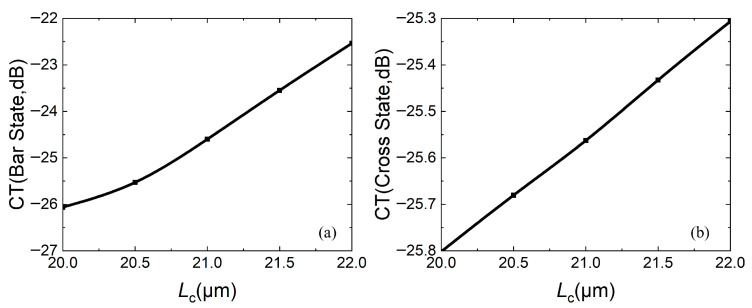
Relationship between crosstalk CT and coupling length *L*_c_: (**a**) bar state; (**b**) cross state.

To further determine the coupling length, the relationship between the coupling length and insertion loss (IL), extinction ratio (ER), and crosstalk (CT) is calculated, as shown in Figure 7, Figure 8 and Figure 9, respectively. For insertion loss, the smaller the better (expressed as an absolute value), whether the device is in the bar state or the cross state. Figure 7a shows that as *L*_c_ increases, insertion loss firstly increases and then decreases, while Figure 7b shows that as *L*_c_ increases, the insertion loss decreases. In both states, the insertion losses are less than 0.5 dB.

For the extinction ratio, the larger, the better. The larger the ER is, the better performance of the same port in switching light “on” and “off”. Figure 8a,b both show that as *L*_c_ increases, the ER decreases. In both states, the ERs of two ports is greater than 22 dB.

For the crosstalk, the smaller, the better. The smaller the CT is, the smaller the light energy of the port that should not exist under the same state. Figure 9a,b both show that the CT increases with increasing *L*_c_. In both states, two-port CTs are less than −22 dB.

Considering comprehensively, the coupling length *L*_c_ is selected as 21 μm.

**Figure 10:**
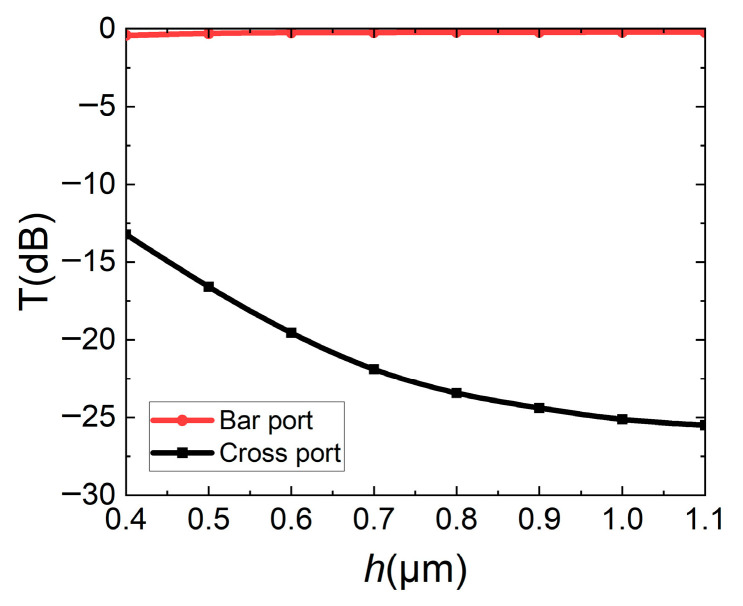
Effect of straight waveguide suspended height *h* on transmissions of bar/cross ports.

In order to further determine that the straight waveguide will not interfere with optical coupling in the bar state when it is suspended, the influence of the straight waveguide suspension height *h* on the transmissions of two output ports is calculated, and the results are shown in Figure 10. It can be seen that when the straight waveguide is suspended by 0.4 μm, the straight waveguide still has a height of 0.1 μm between the DC, causing some light to couple. As the straight waveguide suspended height gradually increases, it is difficult for optical coupling to occur between DC waveguides, the energy of the cross port gradually decreases, and the switch is in the bar state. According to the simulation results, the suspended height *h* of the straight waveguide needs to be higher than 1.1 μm, at which time the suspended straight waveguide will not affect the bar state.

**Figure 11:**
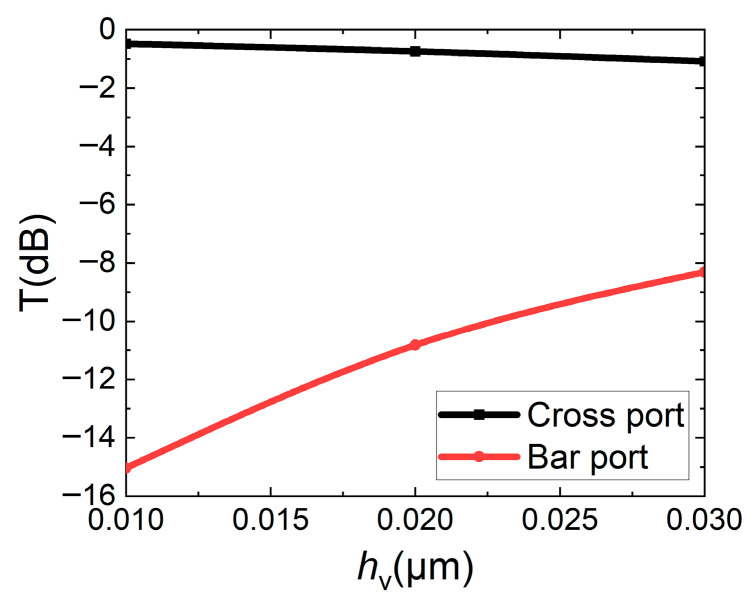
Effect of stitched slot *h*_v_ of straight waveguide stitched to lower cladding layer on transmissions of bar/cross ports.

When the straight waveguide is pulled down, since the surface of the lower cladding layer is possibly rough, the straight waveguide may not be closely stitched to the lower cladding layer. Considering the capacity of the computer and saving calculating time, the mesh in the *Z* direction is set to 0.01 μm, the analysis for the cases where stitched slot *h*_v_ measures 0.01 μm, 0.02 μm, and 0.03 μm between the straight waveguide and the lower cladding is shown in Figure 11. It has the same trend as Figure 10, and failure to closely stitched to the lower cladding layer will seriously affect performance. When the stitched slot is 0.01 μm, the transmissions of the cross port and bar port are 0.48 dB and 15.03 dB, respectively. Chemical mechanical polishing (CMP) needs to be used to flatten the surface of the lower cladding layer in later fabrication. At the same time, when fabricating the straight waveguide, it is also necessary to ensure that the lower surface of the waveguide is flat enough.

**Figure 12:**
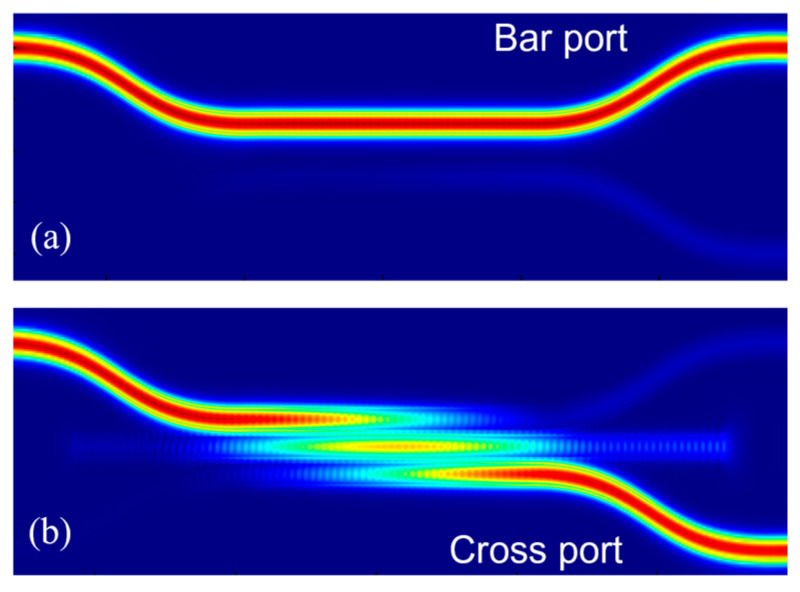
The distribution of optical field under different operating states: (**a**) bar state; (**b**) cross state.

Based on the optimization and confirmation of the above structural parameters, the overall performance is calculated. The optical field distribution in the bar state and cross state are shown in Figure 12a,b, respectively. The structural parameters and key performance are summarized in Table 1. It can be seen that the optical switching function is realized by affecting DC coupling through the straight waveguide. In the bar and cross states, the insertion loss is approximately 0.24 dB and 0.33 dB, the extinction ratio is approximately 24.70 dB and 25.46 dB, and the crosstalk is approximately −24.60 dB and −25.56 dB, respectively. The device shows good optical switch performance.

**Figure 13:**
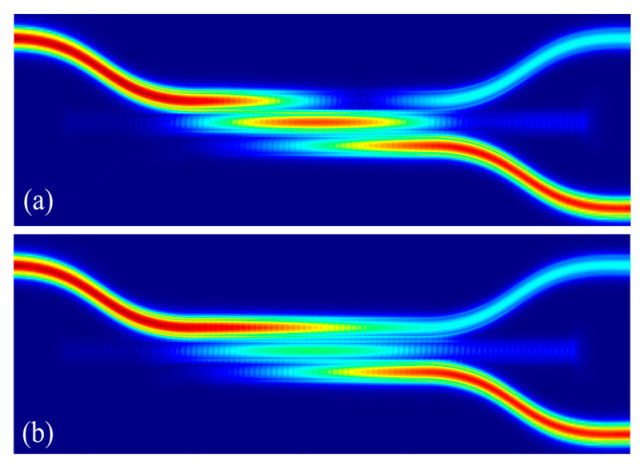
Positioning the straight waveguide down position, distribution of optical field with different offset: (**a**) upward offsets 0.05 μm; (**b**) downward offsets 0.05 μm.

By positioning the straight waveguide at up and down positions in the *Z* direction, it is possible that the straight waveguide will deviate on one side of the DC waveguide in the *X* direction. Figure 13a,b analyze an optical field comparison of the straight waveguide at the down position with an upward and downward offset 0.05 μm of the DC. When the straight waveguide is offset by 0.05 μm upward, the coupling length between the DC input waveguide and the straight waveguide remains unchanged, but the coupling gap decreases, so some of the light is coupled back to the DC waveguide. At this time, the transmission of the bar port is 7.90 dB and the transmission of the cross port loss is 1.08 dB. When the straight waveguide is offset by 0.05 μm downward, similarly, the coupling gap increases, so some of the light is not coupled into the straight waveguide. At this time, the transmission of the bar port is 7.98 dB and the transmission of the cross port is 1.07 dB.

**Figure 14:**
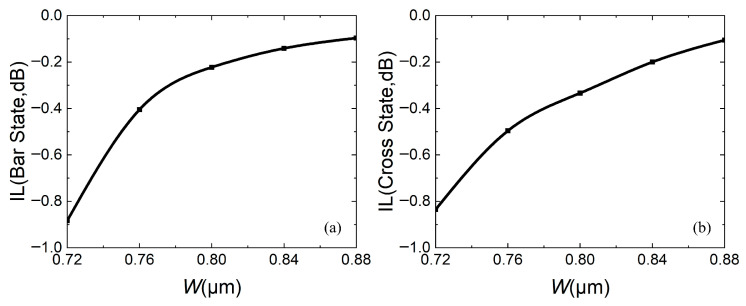
When fabrication error is ±5% and ±10%, relationship between insertion loss and waveguide width *W*: (**a**) bar state; (**b**) cross state.

**Figure 15:**
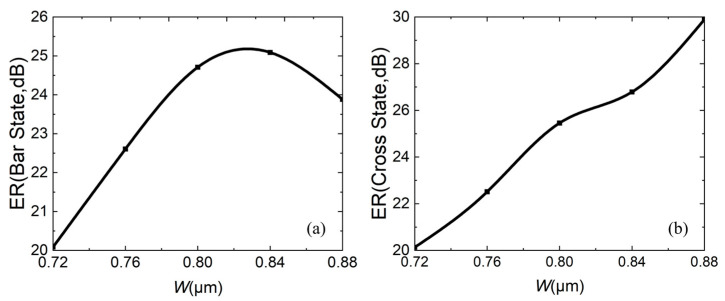
When fabrication error is ±5% and ±10%, relationship between extinction ratio ER and waveguide width *W*: (**a**) bar state; (**b**) cross state.

**Figure 16:**
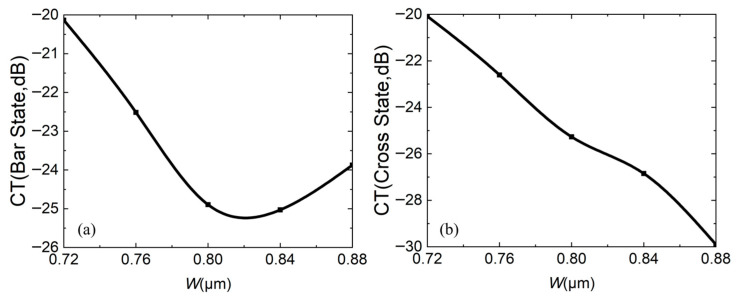
When fabrication error is ±5% and ±10%, relationship between crosstalk CT and waveguide width *W*: (**a**) bar state; (**b**) cross state.

At the same time, fabrication errors are also calculated. Figure 14, Figure 15 and Figure 16 analyze the impact of fabrication errors at ±5% and ±10% on insertion loss, extinction ratio, and crosstalk. It should be noted that the following simulations are performed under the conditions that the waveguide width increases and the coupling gap decreases, while the waveguide width decreases and the coupling gap increases. Figure 14a,b shows the insertion loss in the bar and cross states, respectively. It can be seen that the insertion loss decreases with increasing waveguide width, due to an increase in the mode restriction ability, and the bar state leads to reduced insertion loss. The cross state is such that the coupling efficiency between the waveguides is higher at a wider waveguide width, and there is less residual light in the straight waveguide, which leads to reduced insertion loss. If device insertion losses are less than 0.5 dB in both states, the waveguide width must be wider than 0.76 μm. Figure 15a,b are the extinction ratios of the bar port and cross port, respectively, and Figure 16a,b are the crosstalk of the bar port and cross port, respectively. From Figure 15 and Figure 16, a fabrication error of ±5% and ±10% can satisfy an extinction ratio greater than 20 dB and a crosstalk of less than −20 dB, which shows that the fabrication tolerance of the device is large. In addition, when the fabrication error is +5%, corresponding to *W* = 0.84 μm and *g* = 0.21 μm, it has the best comprehensive performance.

**Figure 17:**
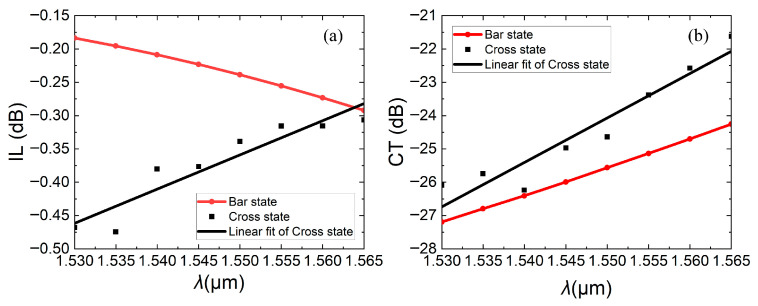
When operation wavelength *λ* is at 1.530~1.565 μm, (**a**) insertion losses and (**b**) crosstalk varies with *λ* in bar and cross states.

In the field of LiDAR, phase modulation can be used for horizontal scanning, and frequency (or wavelength) modulation can be used for vertical scanning. In the field of optical communications, wavelength division multiplexing (WDM) technology is often used to increase data capacity. Therefore, the optical switch needs to work with low insertion loss within a certain optical bandwidth. Figure 17a,b calculates the insertion losses and crosstalk in the C band.

When the device is in the bar state, the straight waveguide is suspended. As the wavelength increases, the insertion loss increases, because of the material’s dispersion effect. As the wavelength increases, the device’s effective refractive index diminishes. Consequently, the waveguide’s capacity to confine light weakens, leading to heightened waveguide propagation loss. Simultaneously, the coupling length required for complete coupling shortens. Given that the actual coupling length remains fixed, a greater portion of light is coupled into the cross port, thereby resulting in increased insertion loss and crosstalk at the bar state.

When the device is in the cross state, the straight waveguide is pulled down. The same principle applies as above: the required coupling length for complete coupling is reduced, and the residual light of the straight waveguide is decreased because of shortened complete coupling and it is better coupled to the cross port. Also due to the above reason, a greater portion of light is coupled into the bar port again. Light in the cross state is coupled twice, and insertion loss and crosstalk do not change completely linearly with wavelengths, as shown in the square black dots in Figure 17. After linear fitting, they are shown as a solid black line. As a result, the insertion loss of cross states is reduced and crosstalk enhances with increasing wavelength.

## 4. Potential Fabrication Route

**Figure 18:**
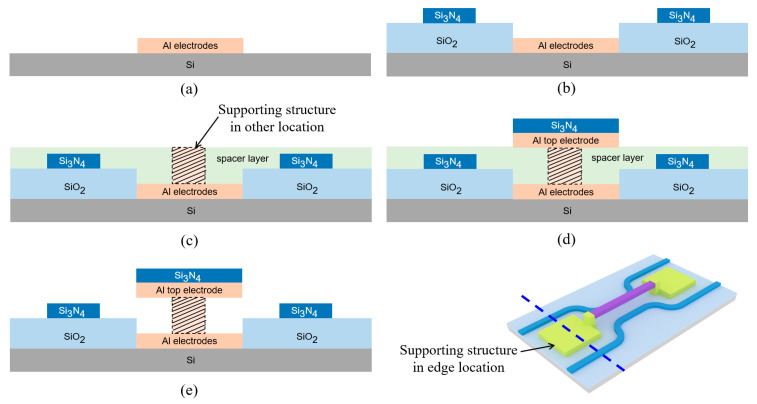
Fabrication route of the proposed optical switch. (**a**) Deposit Al and etch two electrodes. (**b**) Deposit SiO_2_ and Si_3_N_4_ as the lower cladding layer and core layer of the DC structure. (**c**) Deposit the spacer layer, etch a groove and fill the groove with Al for supporting structure. (**d**) Deposit Al and Si_3_N_4_ as the top electrode located at the suspended position, and form the straight waveguide, simultaneously. (**e**) Release the spacer layer.

The fabrication verification of a new design costs a lot. Here, we design a potential fabrication route for the proposed optical switch in Figure 18. In the inset at the bottom right of Figure 18, the blue dashed line represents the cross-section described in the process flow. The total process flow is as follows: (a) Deposit metal aluminum (Al) and selectively etch two electrodes. The two Al electrodes are the top electrode and bottom electrode, respectively, and they are not conductive to each other. (b) Deposit SiO_2_ and Si_3_N_4_ as the lower cladding layer and core layer of the DC, respectively. It is necessary to ensure the surface of the SiO_2_ layer is flat, and CMP should be used. Expose the two Al electrodes at the same time. (c) Deposit the spacer layer and etch a groove for the supporting structure on the Al top electrode located at the lower position. The spacer layer material can be polysilicon, photoresist, etc. Fill with Al to form a supporting structure for electric connection between the two top electrodes. (d) Deposit Al and Si_3_N_4_ as the top electrode located at the suspended position. Here, Si_3_N_4_ is used to enhance the stress of suspended top electrode. The straight waveguide is also formed, simultaneously. (e) Release the spacer layer. Here, the total process is finished. If the spacer layer material is photoresist, oxygen plasma can be used. If the spacer layer material is polysilicon, wet etching can be used. It can be seen that the above processes are compatible with PIC technology.

Actually, the fabrication in Figure 18 is a planar process. It is very easy for high-volume manufacturing and cascading hundreds and thousands of optical switch units to construct a switch array. For example, optical switch units are cascaded to different network architectures according to blocking characteristics, crosstalk suppression, total number of switching units, and number of cascade stages. Some classic switching architectures include Crossbar [17], Banyan [11], Benes [29], PILOSS networks [30], etc. Only for network architecture, the proposed optical switch is not different from the typical thermo-optic switch or electro-optic switch, except in the mechanism of realizing the switching function, so it is also applicable to these architectures.

## 5. Feasibility and Mechanical Analysis

The operating mechanism is that the electrodes on the Si layer provide the different voltages, the electric potential difference in the two electrodes leads to electrostatic force, and the straight waveguide is pulled down. Due to the thickness of the SiO_2_ layer being higher, the straight waveguide touches the SiO_2_ lower cladding layer and stops. Two electrodes cannot touch. When there is no potential difference, the electrostatic force disappears. Due to the Si_3_N_4_ film deposited on the suspended Al top electrode to enhance the stiffness of the Al film, a suitable voltage is used without destroying the suspended electrode structure, and the electrodes can rebound to their initial position by relying on inherent stress. Stiction is less likely to occur. In addition, the suspended Si_3_N_4_ straight waveguide has a large aspect ratio, but there is no need to worry about deformation. Similar structures are referenced in [31,32].

Although the electrodes are located near the waveguides, the device is essentially unaffected by heat. This is due to the following reasons: Firstly, both Si_3_N_4_ and SiO_2_ are materials with low thermo-optic coefficients. Secondly, the waveguide is situated above the SiO_2_ lower cladding layer, and SiO_2_ is a material with low thermal conductivity; above the electrodes is air, whose thermal conductivity is even poorer than that of SiO_2_. Thirdly, there is a certain distance between the electrode and the waveguide, and the electrodes are in direct contact with the Si layer, and Si serves as an excellent heat sink. Fourthly, the switch is actuated by electrostatic attraction induced by potential difference, where the voltages of the upper and lower electrodes is different. There is no directly conducting current and no metal wires with high resistance, resulting in minimal heat generation.

For the proposed electrostatically actuated optical switch, the actuation voltage characteristics of cross and bar states are inherently distinct. Cross-state operation requires a sustained voltage to maintain the electrostatic attraction between the top and bottom electrodes, ensuring the waveguide adheres to the lower cladding. Bar state operation relies on the elastic recovery of the suspended electrode structure after the voltage is removed, requiring no additional power input. According to the structure and mechanism of the proposed switch, the stability will not be a problem and there is reliable feasibility.

The pull-in voltage *V*_*pi*_ of electrostatic parallel plate actuators is described by Equation (1) [33]. The key parameters include the stiffness *k*, the overlap area *A* of the parallel electrodes, and the initial gap *d*_0_ between the electrodes. ε is the permittivity of air between the electrodes. It can be seen that *V*_*pi*_ is positively correlated with *k* and *d*_0_ and negatively correlated with ε and *A.*

(1) $$V_{pi}=\sqrt{\frac{8kd_{0}^{3}}{27\varepsilon A}}$$

According to Equation (1) above, the ε of air is 8.85 × 10^−12^ F/m, based on the results of Figure 10, and assuming *d*_0_ is 1.1 μm, *A* is 54 μm^2^. The key factor is the *k* of the suspended electrode material. In our design, the suspended top electrode material is hybrid, and the Si_3_N_4_ is the key element that influences the stiffness. In simplified models and calculations, only Si_3_N_4_ is considered. The factor *k* that electrostatically actuates planar capacitive actuators with a single support structure for an edge is simplified to Equation (2):

(2) $$k\;=\;\frac{{Ewt}^{3}}{4l^{3}}$$

Assume the suspending Si_3_N_4_ film has 0.1 μm thickness *t*, 9 μm length *l*, and 6 μm width *w.* The Young’s modulus *E* of Si_3_N_4_ is 290 GPa, so via Equation (2), *k *≈ 0.6 N/m. Then, via Equation (1), *V*_*pi*_ ≈ 22.3 V.

As for one proposed structure, the resonant frequency *f* can be calculated by Equation (3). *m* is the mass of one side-suspended structure. It consists of two parts: one is the mass *m*_1_ of the electrode, and the other is the mass *m*_2_ of the straight waveguide. *m*_1_ = ($\rho_{\mathrm{Al}}+\rho_{{\mathrm{Si}}_{3}N_{4}}$) × *lwt* = 3.2 × 10^−14^ kg, and *m*_2_ = 6.4 × 10^−14^ kg. However, the weight of the straight waveguide is borne by the two electrodes, *m* = *m*_1_ + 0.5 *m*_2_, with *f* ≈ 0.5 MHz.

(3) $$f=\frac{1}{2\pi }\sqrt{\frac{k}{m}}$$

The resonant frequency is the natural frequency of the material and structure under specific conditions. If a device operates at this frequency, it will malfunction. Therefore, to ensure normal operation, the device’s working frequency should be less than 1/10 of the resonant frequency. Theoretically, higher resonant frequencies correspond to faster switching speeds. A rough estimate of the switching speed is the reciprocal of the resonant frequency [34]. Based on this assessment, the switching speed may be 20 μs. Actually, switching speed is included the cross and bar times. The factors affecting the two switching times are also different. The cross time (pull-down) is mainly affected by stiffness. As for the bar time, it depends on the suspended structure’s built-in tension to snap it back to the suspended position. Damping action of air, stiffness and tension of films, and suspended height are all key factors.

## 6. Conclusions

In this paper, a new type of optical switch is presented, which can be enabled by an actuated straight waveguide up and down in the middle of a DC waveguide. In other words, this optical switch can be performed by using the straight waveguide to affect optical coupling between two arms of the waveguides in the DC structure, and it realizes the switching optical route. Compared with traditional technologies, this device does not require heating nor introduce carriers. Here, the 3D FDTD method is used to optimize structure parameters in detail, and device performance is also calculated and discussed. It is found that for a device size of 8.8 μm × 55 μm, the insertion losses in the bar and cross state are approximately 0.24 dB and 0.33 dB, the extinction ratios are approximately 24.70 dB and 25.46 dB, and the crosstalks are approximately −24.60 dB and −25.56 dB, respectively. In the C band of optical communication, the insertion loss ranges from 0.18 dB to 0.47 dB. At the same time, the effects of fabrication offset error of waveguides on device performance are also simulated and analyzed. The optical switch proposed in this paper, with its small footprint, low insertion loss, high extinction ratio, low crosstalk, and broad bandwidth in the C band, provides an efficient and reliable solution for optical signal switching in optical communications, data centers, LiDAR, and other fields, and it also provides important reference and support for the development of related technologies.

## Figures and Tables

**Table 1:** Summary of the optimized switch structure parameters and key performance.

**Parameters**	**Value (μm)**	**Loss (dB)**	**ER (dB)**	**CT (dB)**
*W*/*w* × *H*	0.8 × 0.5	0.24 (Bar), 0.33 (Cross)	24.70 (Bar), 25.46 (Cross)	−24.60 (Bar), −25.56 (Cross)
*L*_s-y_	2.9			
*L*_s-x_	17			
*G*	1.3			
*g*	0.25			
*L*_c_	21			

## Data Availability

The dataset is available on request from the authors.
